# Efficacy and Safety of Inhaled Carbon Monoxide during Pulmonary Inflammation in Mice

**DOI:** 10.1371/journal.pone.0011565

**Published:** 2010-07-13

**Authors:** Michael R. Wilson, Kieran P. O'Dea, Anthony D. Dorr, Hirotoshi Yamamoto, Michael E. Goddard, Masao Takata

**Affiliations:** Anaesthetics, Pain Medicine and Intensive Care, Imperial College London, Chelsea and Westminster Hospital, London, United Kingdom; University of Giessen Lung Center, Germany

## Abstract

**Background:**

Pulmonary inflammation is a major contributor to morbidity in a variety of respiratory disorders, but treatment options are limited. Here we investigate the efficacy, safety and mechanism of action of low dose inhaled carbon monoxide (CO) using a mouse model of lipopolysaccharide (LPS)-induced pulmonary inflammation.

**Methodology:**

Mice were exposed to 0–500 ppm inhaled CO for periods of up to 24 hours prior to and following intratracheal instillation of 10 ng LPS. Animals were sacrificed and assessed for intraalveolar neutrophil influx and cytokine levels, flow cytometric determination of neutrophil number and activation in blood, lung and lavage fluid samples, or neutrophil mobilisation from bone marrow.

**Principal Findings:**

When administered for 24 hours both before and after LPS, inhaled CO of 100 ppm or more reduced intraalveolar neutrophil infiltration by 40–50%, although doses above 100 ppm were associated with either high carboxyhemoglobin, weight loss or reduced physical activity. This anti-inflammatory effect of CO did not require pre-exposure before induction of injury. 100 ppm CO exposure attenuated neutrophil sequestration within the pulmonary vasculature as well as LPS-induced neutrophilia at 6 hours after LPS, likely due to abrogation of neutrophil mobilisation from bone marrow. In contrast to such apparently beneficial effects, 100 ppm inhaled CO induced an increase in pulmonary barrier permeability as determined by lavage fluid protein content and translocation of labelled albumin from blood to the alveolar space.

**Conclusions:**

Overall, these data confirm some protective role for inhaled CO during pulmonary inflammation, although this required a dose that produced carboxyhemoglobin values close to potentially toxic levels for humans, and increased lung permeability.

## Introduction

Neutrophilic pulmonary inflammation is a major contributor to morbidity in a variety of both acute and chronic respiratory disorders. Despite much research, therapies to modulate the inflammatory cascade and ensuing pulmonary dysfunction that are efficacious, safe and readily manipulable have been elusive.

Recently, many experimental studies have shown a beneficial effect of low dose inhaled carbon monoxide (CO) on the progression of various types of tissue injury, with CO having anti-inflammatory, antifibrotic, and antiapoptotic effects [1]. Inhaled agents clearly have great potential for the treatment of pulmonary disorders, and studies have shown efficacy of CO (typically 250–1000 parts per million (ppm)) using in vivo models of lung injury and inflammation induced by bleomycin [2], aeroallergens [3], mechanical stretch [4], ischemia-reperfusion [5], [6], hyperoxia [7] and acid aspiration [8]. However, there have been several animal studies that did not find beneficial impacts of inhaled CO [9]–[12]. Furthermore, in the human studies carried out to date (involving healthy volunteers and stable chronic obstructive pulmonary disease (COPD) patients), no significant benefits of inhaled CO to attenuate pulmonary inflammation were observed [13], [14]. These apparent inconsistencies may reflect gaps in our knowledge of the mechanisms of CO action, potentially leading to inappropriate choices of models/clinical subjects, dosing regimens, measured output variables etc. Alternatively, this may indicate that the efficacy of CO is limited to particular species and/or etiologies of injury/inflammation. Despite the initially impressive pre-clinical results, considerable work clearly still needs to be carried out to improve the chances of designing efficacious and most importantly safe therapies using CO.

The aim of the current study was therefore to systematically investigate the impact of CO, including anti-inflammatory effects, safety, efficacy, dosing requirements and mechanisms of action within a simple model, to help shed light on the reasons behind the inconsistencies within the literature. We previously found no beneficial effect of CO in various models of severe acute lung injury in mice, induced by high doses of lipopolysaccharide (LPS) or oleic acid [10]. This may be due to the rapidly progressing (lasting 2–3 hours) and overwhelming nature of these injury models. For the current study we therefore utilised a mouse model of moderate pulmonary inflammation and barrier permeability induced by a low dose intratracheal LPS administration. The data show that 100 ppm inhaled CO was effective to reduce neutrophil recruitment to the lungs, via a novel mechanism linked to decreased bone marrow mobilisation of leukocytes. However even this dose of CO, which is lower than generally used in pre-clinical studies, seemingly led to increased pulmonary endothelial/epithelial barrier permeability, suggesting that while CO could potentially be effective in treating pulmonary inflammation and lung injury, the therapeutic window may be small.

## Materials and Methods

### Intratracheal LPS-induced pulmonary inflammation model

All experimental protocols involving animals were approved by the Ethical Review Board of Imperial College London, and carried out under the authority of the UK Home Office in accordance with the Animals (Scientific Procedures) Act 1986. Male C57Bl/6 mice (Charles River, Margate, UK) aged 8–12 weeks were anesthetised with ketamine (60 mg/kg) and xylazine (6 mg/kg). Animals were suspended in the upright position and an external light source used to illuminate the larynx. A fine polyethylene catheter (external diameter 0.61 mm, internal diameter 0.28 mm) was then passed into the trachea via the mouth under direct visualisation of the vocal cord, using an adaptation of previously described methods [15]. 10 ng LPS (Ultrapure LPS, InVivoGen) in 50 µl saline was administered into the trachea, and animals were allowed to recover on a heated bed. After 6 or 24 hours mice were sacrificed by pentobarbital overdose, blood samples were obtained by cardiac puncture, and lung lavage was performed with 750 µl saline [16].

### CO exposure

Mice were exposed to air or air/CO before and/or after LPS instillation (for detailed protocols, see Results) in a custom-made gas exposure chamber, fed with continuous gas flows of air and a mix of air/5000 ppm CO (Carburos Metalicos, Madrid, Spain). Animals exposed post-LPS were placed in the chamber as soon as adequate recovery of respiration and body temperature was achieved (typically ∼30–40 minutes after instillation). The flows from each gas mixture were adjusted to give final CO concentrations of 0–500 ppm, with a total flow rate of 900 ml/min to avoid chamber hypoxia/hypercapnia. Animal numbers in the chamber were restricted to four to ensure consistent environmental conditions. Probes within the chamber allowed continuous monitoring of temperature, humidity, CO and CO_2_ levels (Testo 650, Testo Ltd, Alton, UK). O_2_ levels within the chamber were intermittently evaluated.

### Assessment of intraalveolar inflammation

Lavage fluid samples were analysed for protein concentration using BioRad assay reagent (BioRad Laboratories, Hemel Hempstead, UK) and for cytokines including interleukin-6 (IL-6), macrophage inflammatory protein-2 (MIP-2), keratinocyte-derived chemokine (KC) and interleukin-10 (IL-10) using ELISA kits (R&D Systems, Abingdon, UK). Intraalveolar neutrophil infiltration was determined by hemacytometer and differential cytology on lavage cell samples.

### Flow cytometry

In some experiments, neutrophil numbers and activation status in lavage, lung and blood samples were analysed by flow cytometry, as described previously [17], [18]. In brief, single cell suspensions were prepared from excised lungs of mice by mechanical disruption to analyse lung-marginated neutrophils. Lavage, lung and blood cell samples were then stained with fluorochrome-conjugated antibodies against cell-surface markers (CD11b, F4/80, Gr-1, L-selectin) and analysed by flow cytometry (FACSCalibur, Becton Dickinson, Oxford, UK). Microsphere counting beads (Caltag Medsystems, Towcester, UK) were added to enable cell quantification. Neutrophils were identified based on forward/side-scatter properties and F4/80 and Gr-1 expression. Neutrophil activation was assessed by surface expression levels of L-selectin and CD11b.

### Neutrophil mobilisation

In a series of experiments to ascertain the influence of CO on neutrophil mobilisation from bone marrow, mice received an intraperitoneal injection of 0.2 ml BromodeoxyUridine (BrdU, 10 mg/ml in saline) [18]. After 48 hours to allow incorporation of BrdU into dividing leukocytes, mice were administered LPS and exposed to 100 ppm CO as described above. After 6 hours, blood and lung cell suspensions were processed and stained using a BrdU ‘Flow Kit’ according to manufacturer's instructions (BD Pharmingen, Oxford, UK). Cells were stained with appropriate neutrophil marker antibodies and then washed, fixed, permeabilised and treated with DNAse to reveal BrdU epitopes. BrdU incorporation was determined by staining with an allophycocyanin (APC) anti-BrdU monoclonal antibody, and the percentage of neutrophils positive for BrdU was evaluated by flow cytometry.

### Pulmonary barrier permeability

To determine the influence of inhaled CO on pulmonary barrier permeability, mice (without LPS treatment) were exposed to either air or 100 ppm CO in the exposure chamber. After 5 hours, mice received a tail vein injection of 100 µl (0.1 mg) fluorescence-labelled albumin (Alexa-Fluor 594-labelled albumin, Invitrogen) and were returned to the exposure chamber. After one further hour, mice were terminated by anesthetic overdose, and blood and lung lavage fluid samples taken. Permeability was estimated by the ratio of lavage fluid : plasma fluorescence [19].

### CO-Hb association/dissociation in mice

In a separate series of experiments, anesthetised and tracheostomised mice were ventilated with a custom-made mouse ventilator (8–9 ml/kg tidal volume, 2.5 cmH_2_O positive end-expiratory pressure (PEEP), respiratory rate 120), with a carotid arterial line inserted for blood sampling, fluid infusion (0.4 ml/hr saline containing 10 U/ml heparin) and monitoring arterial blood pressure [16]. A polyethylene catheter was introduced into the intraperitoneal cavity for maintenance of anesthesia (ketamine 36 mg/kg : xylazine 3.6 mg/kg bolus every 20–25 minutes). To assess carbon monoxide-hemoglobin (CO-Hb) association kinetics, mice were initially ventilated with air, a blood sample removed for determination of baseline carboxyhemoglobin % (COHb), and then the inspired gas was switched to 500 ppm CO. Ventilation was continued for 80 minutes with blood samples taken every 20 minutes. For dissociation kinetics, a separate set of mice were instrumented and ventilated with 500 ppm CO for 80 minutes, following which a baseline blood sample was taken, the inspired gas changed to air, and ventilation continued for a further 80 minutes with blood sampling every 20 minutes. Blood sample volume (∼70 µl) was replaced with saline following each sampling to ensure stable blood pressure and hemodynamics throughout experiments.

### Statistical Analysis

Data are expressed as mean±SD. Statistical comparisons were made by t-tests or ANOVA with Bonferroni tests, using GraphPad Prism software. Statistical significance was defined as p<0.05.

## Results

### Impact of CO on LPS-induced neutrophil infiltration

**Figure 1:**
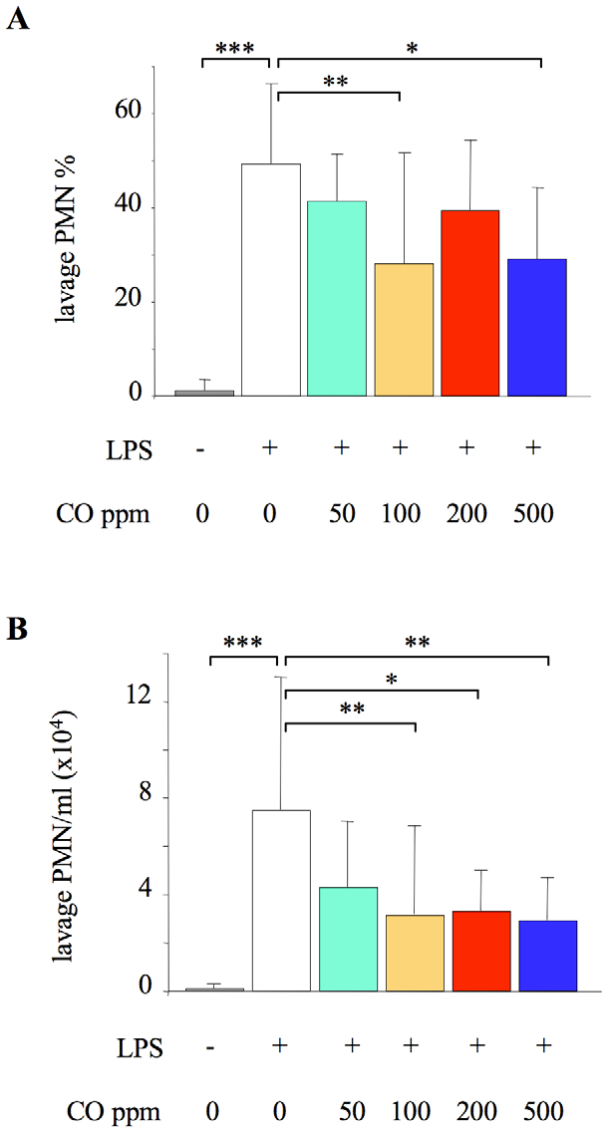
Alveolar neutrophil recruitment 24 hours after LPS. Impact of carbon monoxide (CO) exposure on neutrophil (PMN) percentage (**A**) and number/ml (**B**) in lung lavage fluid of untreated animals (no LPS or CO), or mice treated with 10 ng intratracheal LPS. LPS-challenged mice were exposed to either 0 (air), 50, 100, 200, or 500 ppm CO for 24 hours both before and after LPS. *p<0.05, **p<0.01 ***p<0.001 vs LPS +0 ppm CO; n = 19 for LPS +0 ppm CO, and 8–12 for all other groups (numbers are higher in the LPS+0 ppm CO group because, as our primary control, we ran 1–2 of these animals alongside the experiments for each of the other groups).

No animals died after randomisation to treatment groups in any of the experimental protocols. Initial experiments were carried out using prolonged exposure to CO, to determine the maximal impact of CO both in terms of anti-inflammatory consequences and potential side-effects. Recruitment of neutrophils into the alveolar space was determined in animals exposed to 0–500 ppm CO for 24 hours both before and after LPS (fig. 1). LPS induced a substantial intra-alveolar neutrophil infiltration compared to untreated mice, while inhaled CO of 100 ppm or more significantly reduced this by ∼40–50%.

### Side-effects of CO exposure

**Figure 2:**
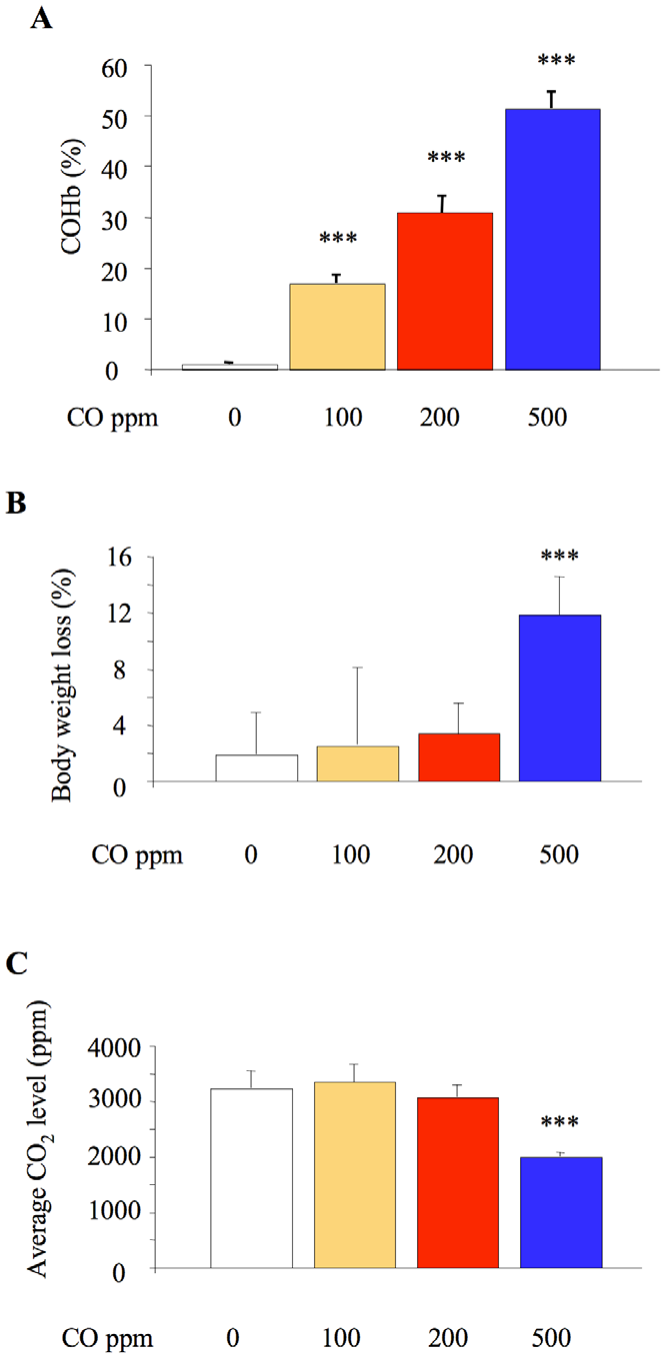
Indicators of side-effects with low dose inhaled carbon monoxide. **A**. Carboxyhemoglobin (COHb) level in blood of animals exposed to carbon monoxide (CO) for 24 hours both before and after lipopolysaccharide (LPS) instillation. Only COHb data from the first mouse removed from the chamber are shown to minimise the confounding effects of dropping the CO concentration upon opening the chamber. ***p<0.001 vs 0 ppm CO, n = 4–5/group. **B**. Percentage weight loss in the 24 hours following LPS instillation, in mice exposed to 0, 100, 200 or 500 ppm. ***p<0.001 vs 0 ppm CO, n = 8–12/group. **C**. CO_2_ level in chamber. CO_2_ levels were recorded every 30 minutes: data represent average level in the 24 hour period prior to LPS instillation (to avoid potential confounding effects of anesthetic/LPS). ***p<0.001 vs 0 ppm CO, n = 3–5 experiments, with 4 mice in the chamber each experiment.

To investigate possible side-effects, COHb levels in cardiac puncture blood were determined in LPS-challenged animals, using a hemoximeter with inbuilt algorithms for mouse blood (OSM3, Radiometer, Crawley, UK). As opening the chamber to remove mice inevitably caused a decrease in atmospheric CO, only the data from the first mouse removed from the chamber were compared (fig. 2A). A clear dose response was apparent with increasing CO, with 500 ppm CO producing >50% COHb. 500 ppm CO was also associated with greater weight loss following LPS instillation (fig. 2B), lower CO_2_ levels within the chamber (fig. 2C) and an observed reduction in physical activity. Because CO_2_ was not present in the gas supplied, chamber levels are a reflection of CO_2_ production by the animals. Taken together, these indicate substantial undesirable side-effects of CO exposure at 500 ppm.

### *In vivo* kinetics of CO-Hb association/dissociation

**Figure 3:**
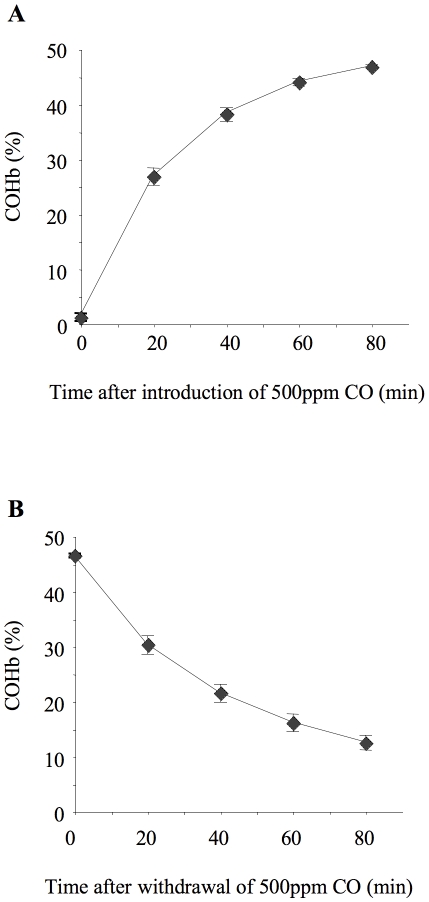
Carboxyhemoglobin association and dissociation kinetics. Time course for association (**A**) and dissociation (**B**) of blood carboxyhemoglobin (COHb) in ventilated, instrumented mice. For association kinetics, mice were ventilated from time 0 with 500 ppm carbon monoxide (CO) and arterial blood samples taken every 20 minutes. For dissociation kinetics a separate set of mice were ventilated for 80 minutes with 500 ppm CO, then at time 0 inspired gas was switched to 0 ppm CO and samples were taken every 20 minutes thereafter. n = 4/time point.

The kinetics of CO-Hb association/dissociation were found to be very rapid in ventilated mice (fig. 3). Blood COHb levels reached 50% of steady-state value (defined as COHb% after 24 hours inhalation) within 20 minutes of exposure, and >90% of steady-state value by 80 minutes. COHb dissociation was similarly rapid, with a half-life of 30–40 minutes after discontinuation of CO.

### Timing of CO exposure

**Figure 4:**
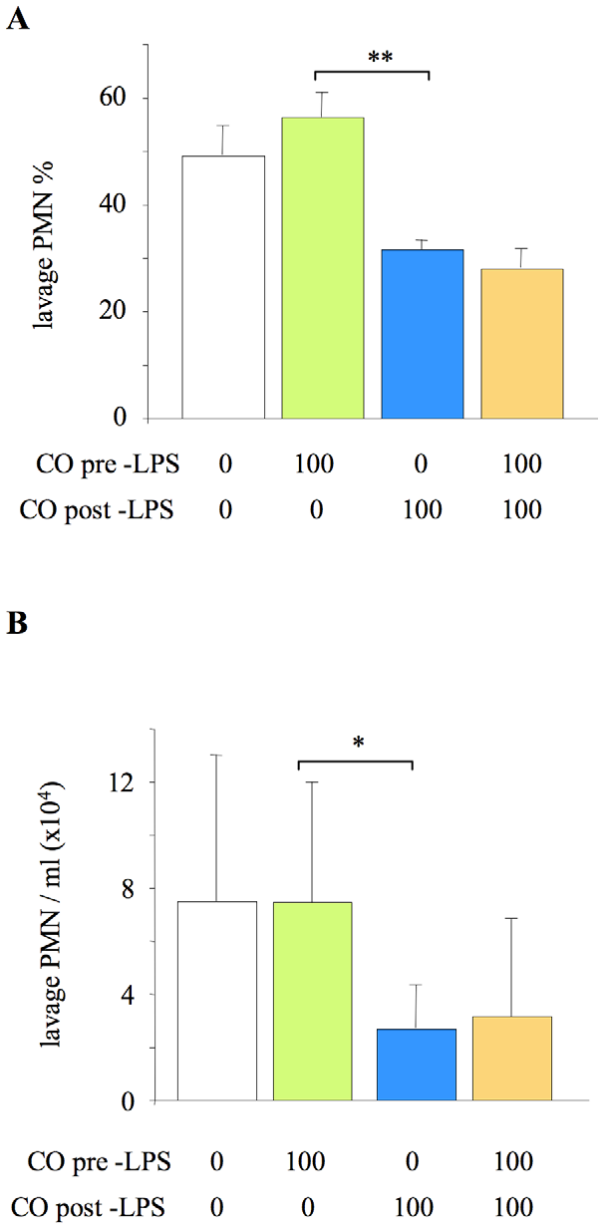
Impact of CO exposure either pre- or post- LPS challenge on alveolar neutrophil recruitment. Neutrophil (PMN) % (**A**) and number/ml (**B**) in lung lavage fluid of mice exposed to 100 ppm carbon monoxide (CO) for 24 hours either before or after lipopolysaccharide (LPS) instillation. *p<0.05, **p<0.01 vs 100 ppm CO pre-LPS; n = 8/group. For comparison, data from Figure 1 of the animals exposed either to 0 ppm or 100 ppm CO for 24 hours both pre- and post-LPS are shown (but not included in statistical analysis).

To determine whether efficacy of inhaled CO required pre-exposure, mice were exposed to 100 ppm CO (as the lowest dose which produced consistent attenuation of neutrophil infiltration) for 24 hours either before or after LPS challenge, and sacrificed for analysis at 24 hours after LPS. The beneficial effect of CO could be attributed entirely to the period following LPS, demonstrating that pre-exposure was unnecessary (fig. 4). Thus, exposure to CO after LPS challenge was utilised for the subsequent experiments.

### Mechanisms underlying attenuated neutrophilic inflammation

**Figure 5:**
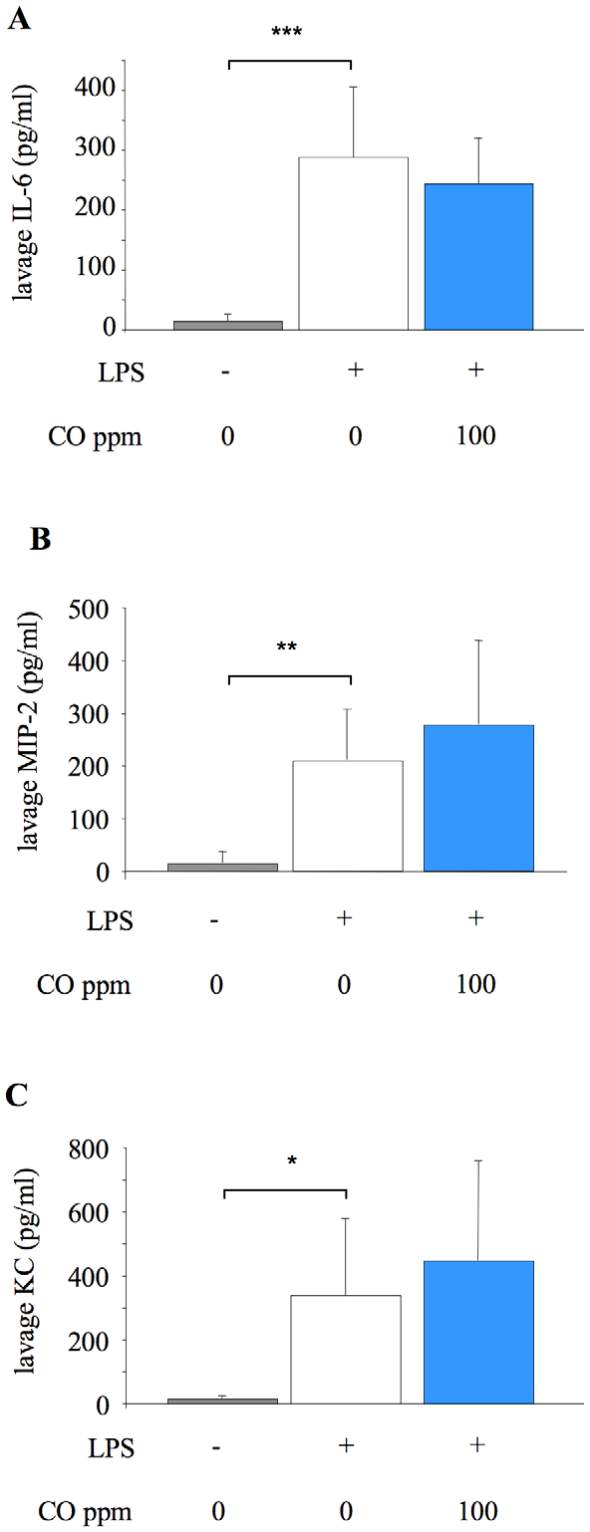
Lavage fluid cytokine concentrations 6 hours after LPS challenge. Concentration of cytokines IL-6 (**A**), MIP-2 (**B**), and KC (**C**) in lung lavage fluid of untreated mice (no LPS or CO), and mice exposed to 0 or 100 ppm carbon monoxide (CO) for 6 hours after LPS instillation. *p<0.05, **p<0.01 ***p<0.001 vs LPS +0 ppm CO; n = 7–8/group for IL-6 and MIP-2; n = 14–15/group for KC.

To examine whether the observed decrease in neutrophil infiltration into the alveolar space following inhaled CO was related to local production of inflammatory mediators, levels of the proinflammatory cytokines IL-6 and MIP-2, and the anti-inflammatory cytokine IL-10 were assessed in lavage fluid of mice exposed to 100 ppm CO for 24 hours after LPS instillation. The level of each of these mediators, with or without CO, was effectively negligible, suggesting that inflammatory mediator response within the alveoli had passed its peak and already returned to baseline by this point in the model. We therefore studied lavage fluid levels of cytokines in mice exposed to 100 ppm CO at 6 hours after LPS instillation. At this earlier time point in the progression of pulmonary inflammation, much higher levels of the proinflammatory mediators (including the addition of KC) were detected, although IL-10 levels were again negligible. There was however no effect of CO exposure on any of the mediators studied (fig. 5).

**Figure 6:**
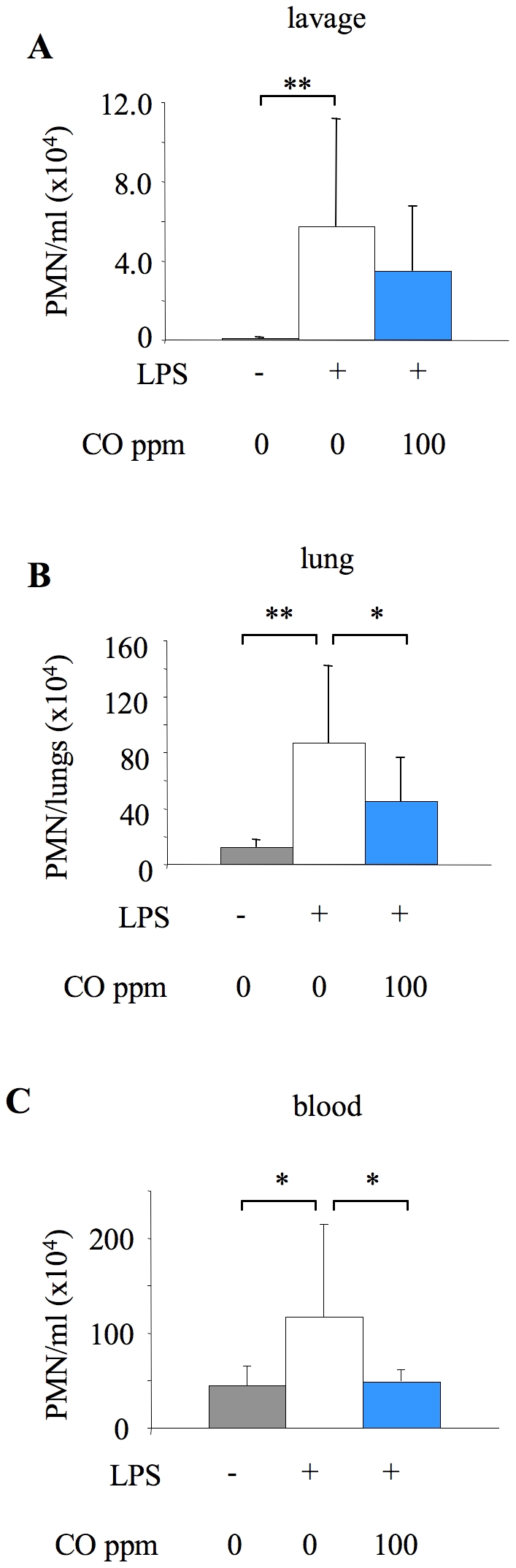
Tissue neutrophil numbers 6 hours after LPS, determined by flow cytometry. Neutrophil (PMN) number in lavage fluid (**A**), lung tissue (**B**) and blood (**C**) from untreated mice (no LPS or CO), or mice exposed to 0 or 100 ppm carbon monoxide (CO) for 6 hours after LPS instillation. Single cell suspensions were prepared from excised lungs of mice by mechanical disruption. Lavage, lung and blood cell samples were stained with fluorochrome-conjugated antibodies against cell-surface markers (CD11b, F4/80, Gr-1, L-selectin) and analysed by flow cytometry. Microsphere counting beads were added to enable cell quantification. Neutrophils were identified based on forward/side-scatter properties and F4/80 and Gr-1 expression. *p<0.05, **p<0.01 vs LPS +0 ppm CO; n = 9–10/group.

**Figure 7:**
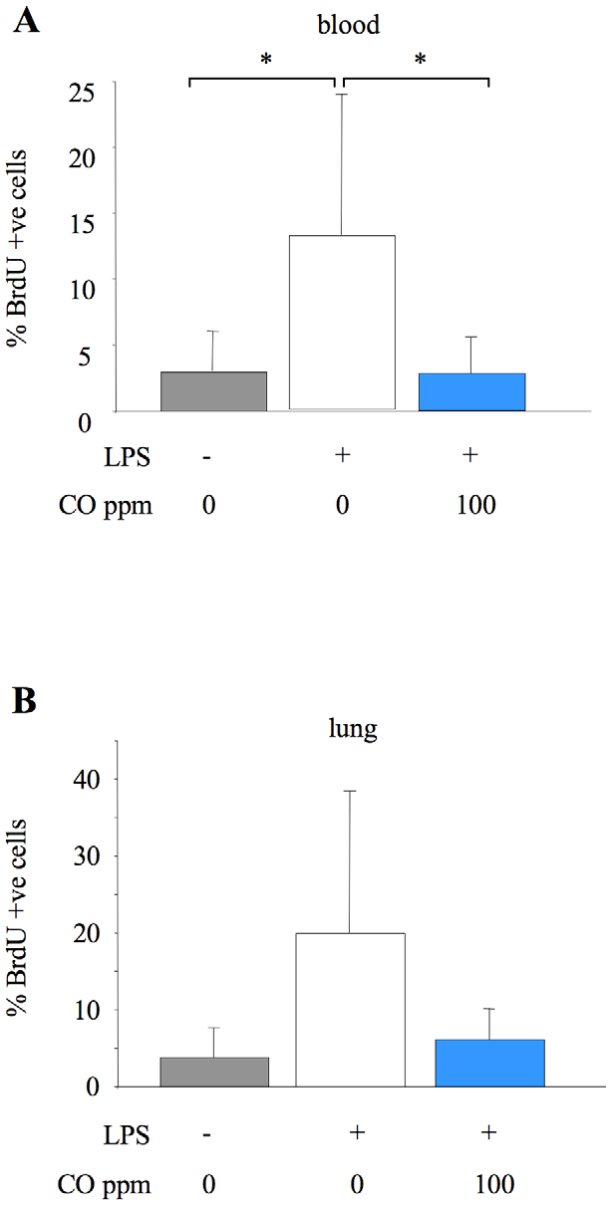
Neutrophil mobilisation. Percentage of newly released BrdU containing neutrophils in blood (A) and lung tissue (B) from untreated mice (no LPS or CO), or mice exposed to 0 or 100 ppm carbon monoxide (CO) for 6 hours after LPS instillation. Neutrophils were identified as described previously, and BrdU incorporated into DNA was detected by flow cytometry using an APC-labelled anti-BrdU antibody. Data are expressed as percentage of neutrophils within tissue positive for BrdU staining. *p<0.05 vs LPS +0 ppm CO; n = 6–7/group.

As the attenuation of alveolar neutrophilia was seemingly not associated with changes in soluble mediator levels, we investigated whether CO influenced early neutrophil activation and sequestration within the lung microvasculature, which has been shown to peak between 4–12 hours following intratracheal LPS [20]. This was assessed using flow cytometry to determine neutrophil numbers and adhesion molecule expression in blood, lung tissue and lavage fluid. 6 hours of LPS treatment induced a significant increase in neutrophil numbers in each of these compartments. 100 ppm CO tended to reduce neutrophil numbers in lavage fluid, although this was not significant at this early point (fig. 6A). Neutrophil sequestration within the lung tissue was however significantly attenuated by CO (fig. 6B). Similarly, the increase in blood neutrophils following LPS was almost completely abrogated by CO (fig. 6C). To determine whether this latter finding was related to an effect of CO to inhibit neutrophil mobilisation from bone marrow (as opposed to other ‘marginated’ pools), mice were dosed with BrdU 48 hours before LPS administration. LPS induced a clear increase in the number of BrdU-containing (i.e. newly released from bone marrow) neutrophils in the blood at 6 hours, which was significantly attenuated by 100 ppm CO exposure (fig. 7). A similar pattern was observed in the lung although changes were not significant.

**Figure 8:**
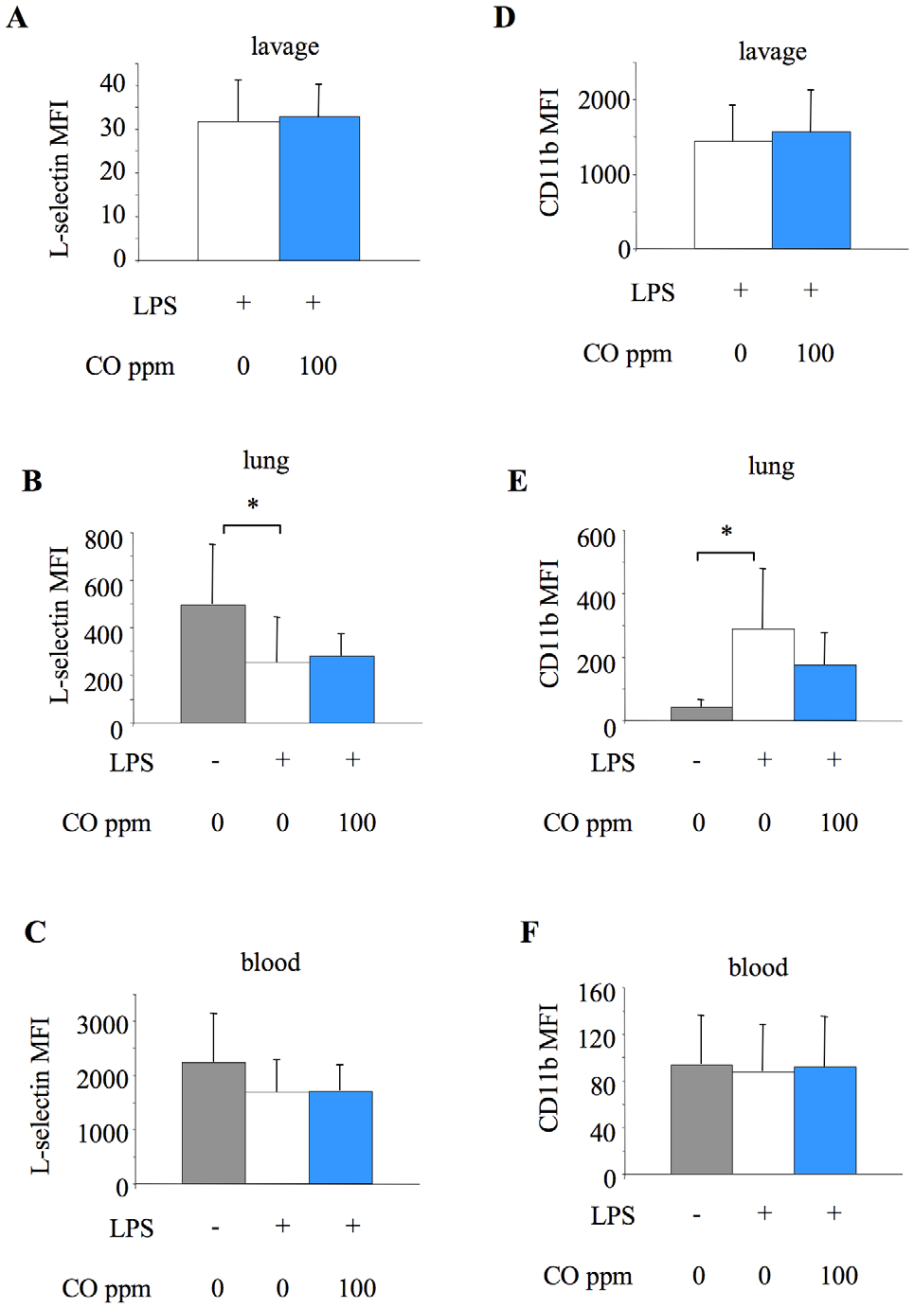
Neutrophil adhesion molecule expression, determined by flow cytometry. Surface expression of L-selectin (**A–C**) and CD11b (**D–F**) on neutrophils from lavage (**A,D**), lung tissue (**B,E**) and blood (**C,F**) from untreated animals (no LPS or CO), or mice exposed to 0 or 100 ppm carbon monoxide (CO) for 6 hours after LPS instillation. Data are expressed as mean fluorescence intensity (MFI). The data of lavage neutrophils in untreated animals were not included because the numbers of cells recovered were too small to allow for accurate analysis. *p<0.05 vs LPS +0 ppm CO; n = 9–10/group.

We also investigated potential changes in the levels of neutrophil activation in terms of surface expression of L-selectin and CD11b (fig. 8). LPS induced a significant activation of lung-marginated neutrophils, promoting shedding of L-selectin (i.e. reduced expression), and upregulation of CD11b. CO had no impact on L-selectin expression, but tended to reduce CD11b expression on lung-marginated neutrophils. Neither LPS nor CO had a significant impact on activation of circulating neutrophils.

### Impact of CO on pulmonary barrier permeability

**Figure 9:**
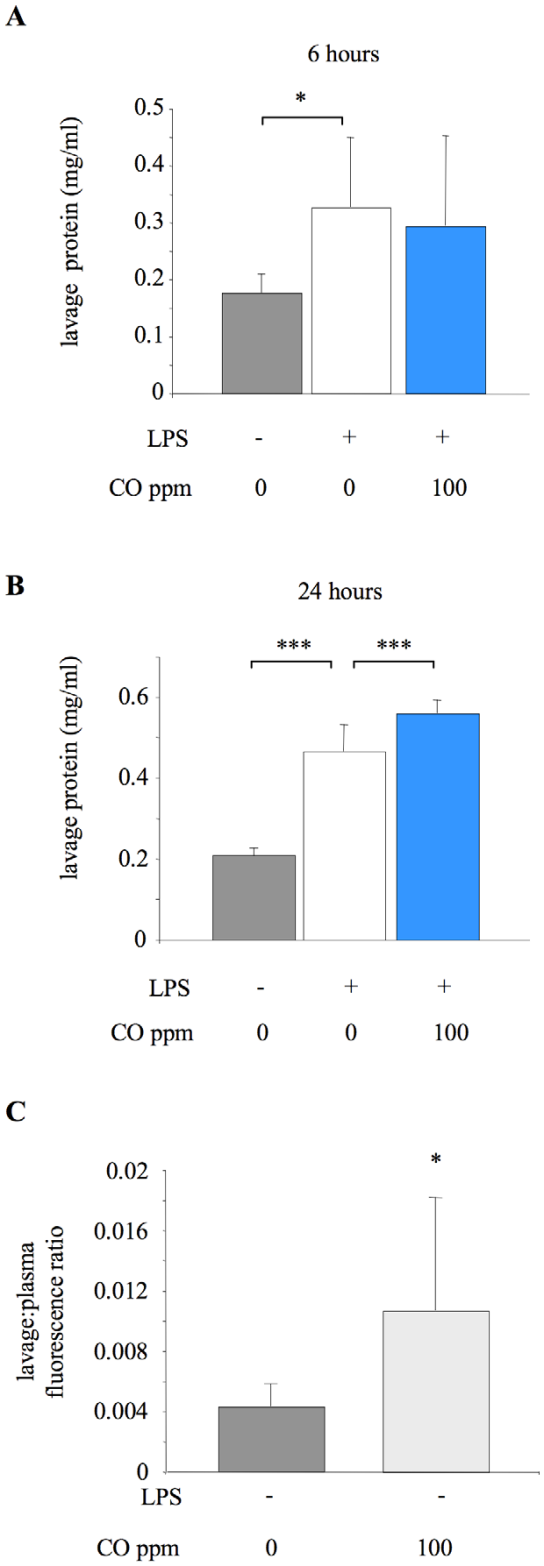
Impact of CO on pulmonary barrier permeability. **A.** Lung lavage fluid total protein concentration from untreated animals (no LPS or CO), or mice exposed to 0 or 100 ppm carbon monoxide (CO) for 6 hours after LPS instillation. *p<0.05 vs LPS +0 ppm CO; n = 8 for untreated animals and 15–16 for LPS treated groups. **B.** Lung lavage fluid total protein concentration from untreated animals or mice exposed to 0 or 100 ppm CO for 24 hours after LPS instillation. ***p<0.001 vs LPS +0 ppm CO; n = 7–8/group. **C.** Permeability was also assessed in both untreated mice (no LPS or CO) and animals receiving 100 ppm CO alone for 6 hours (no LPS) by determining translocation of a fluorescence-labelled albumin from plasma to alveolar space over a 1 hour period. Data are expressed as a ratio of fluorescence between lavage fluid and plasma. *p<0.05 vs untreated group; n = 6–7.

To assess whether the attenuated pulmonary inflammation following CO exposure was associated with a corresponding improvement in pulmonary barrier permeability, we initially determined lavage fluid total protein concentration at 6 and 24 hours after LPS (fig. 9A, B). Lavage fluid protein was significantly increased at both time points after LPS compared to untreated control mice. Somewhat unexpectedly, mice exposed to 100 ppm CO for 24 hours after LPS showed a further increase in total protein. To clarify that this was an effect of CO on barrier dysfunction, permeability was directly assessed in animals exposed to CO alone (i.e. no LPS) for 6 hours. Permeability, in terms of the lavage fluid to plasma ratio of fluorescence-labelled albumin, was significantly enhanced by CO inhalation (fig. 9C).

## Discussion

A number of studies, although not all, have shown protective effects of low dose CO exposure in various animal models of tissue inflammation and dysfunction [21]. As human trials to date have been relatively less successful than these pre-clinical experiments, the current study was designed to investigate the efficacy, safety, timing of administration, and mechanisms of action of low dose inhaled CO in a simple model of pulmonary inflammation and barrier permeability induced by intratracheal LPS instillation. The data demonstrate that CO delivered after the initiating insult has a moderate but significant effect in attenuating LPS-induced pulmonary inflammation. This was associated with decreased neutrophil mobilisation from bone marrow, though produced no improvement but rather some exacerbation in pulmonary permeability.

Previously, we have assessed the impact of inhaled CO on a variety of more severe models of pulmonary inflammation/injury than used here [10]. Intratracheal administration of a high dose of LPS (20 µg) induced substantially greater lavage fluid protein (2–3× greater) and MIP-2 (some 20× greater) than found in the present study, but these were unaffected by CO. In an alternative model of injury induced by intravenous oleic acid, we found extremely high lavage fluid protein levels (∼10× greater than in the current study), associated with substantial deteriorations in respiratory system mechanics and gas exchange, but again no effect of CO on these parameters. In light of these data, the model chosen for this study was designed specifically to produce pulmonary inflammation and increased alveolar epithelial/endothelial permeability of moderate degree, as such changes were felt more likely to be modifiable by low doses of inhaled CO. Although there were no outward clinical manifestations of respiratory distress, the low dose intratracheal LPS instillation used induced substantial increases in alveolar neutrophil infiltration (∼40–60% alveolar leukocytes as neutrophils) and lavage fluid protein (∼0.4–0.45 mg/ml) in these mice. While direct comparisons between animal studies and human patients are difficult, such data are not dissimilar from the lower end of the range reported in human acute lung injury/acute respiratory distress syndrome (lavage neutrophils 70–80%, total protein 0.5–1 mg/ml [22]).

We first exposed mice to 50–500 ppm CO for 24 hours both before and after LPS to explore the maximal likely impact of CO. Intraalveolar neutrophil infiltration was attenuated to a moderate degree (40–50%) by inhaled CO of 100 ppm and above (at 50 ppm any effect of CO was variable and not significant compared to air exposed animals, so was discounted from further investigation). Such changes are less striking than other impressive results reported in the literature for CO, or the effects of more common anti-inflammatory treatments such as glucocorticoids [23], N-acetylcysteine [24] or anti-chemokine antibodies [25]. We did however observe that delivery of CO after the challenge was both necessary and sufficient for efficacy, indicating that prophylaxis may not be a requirement for inhaled CO to be of benefit.

As CO is well known for its toxic effects, it is crucial that animals (and by extension patients) should not be exposed to higher levels than necessary [26]. We therefore carefully evaluated potential side-effects of inhaled CO by examining blood COHb levels as well as any objective clinical signs. We found that 500 ppm CO over 24 hours was clearly toxic, associated with unacceptably high (>50%) blood COHb levels, substantial weight loss and reduced CO_2_ production, presumably reflecting hampered physical activity. Such a dose of CO has previously been reported to induce apoptosis within the brain [9], although over a longer timescale (60 hours) than used currently. Animals exposed to 200 ppm CO showed high (∼31%) blood COHb levels with a tendency, though not significant, to greater weight loss and lower CO_2_ production than mice breathing air. Mice exposed to 100 ppm CO did not show obvious clinical signs so this dose was used for subsequent experiments, although it still induced moderately high blood COHb levels of ∼17%. These levels are around the so-called “biological threshold” for CO tolerance in humans (15–20% COHb), i.e. the levels which in the majority of cases are not detrimental, but beyond which severe CO-mediated injury is likely [27].

The precise mechanisms of action of CO, in particular the downstream ‘physiological’ mechanisms, still remain unclear. The multitude of reported effects include altered cGMP signaling [6], up/down-regulation of signal transduction pathways [4], [28], [29], altered trafficking of toll-like receptors [30] and mediating their interaction with Caveolin-1 [31]. One of the primary physiological end-points of these upstream signal regulation processes is reduced neutrophil infiltration into tissue [4], [29], but how the ‘molecular’ processes affected by CO link to this endpoint is unknown. CO exposure has previously been shown to induce alterations in locally produced cytokines [4], [28], [29], including reduced production of tumor necrosis factor (TNF), IL-6 and IL-1β, and increased production of IL-10. However, in this study CO inhalation had no impact on lavage fluid IL-6, IL-10, or the CXC chemokines MIP-2 and KC. This is somewhat surprising given that the CXC chemokines are major neutrophil chemoattractants, but is consistent with data from a mouse model of acid-induced acute lung injury [8], and isolated RAW 264.7 macrophages exposed to LPS [28].

In contrast, our results demonstrated that inhaled CO attenuated early neutrophil sequestration within the lung vasculature, and almost entirely abrogated LPS-induced neutrophilia. The increased circulating neutrophils following LPS may represent mobilisation of either newly released cells from bone marrow, or cells ‘demarginated’ from other organ beds. Using BrdU to label dividing cells we demonstrated, for the first time to our knowledge, that CO inhibits neutrophil mobilisation from bone marrow (others have suggested that neutrophil activation status may be decreased by CO [8], a finding we did not reproduce here). How this effect of CO on neutrophil mobilisation occurs is unclear - we considered that it may be a consequence of decreased production of granulocyte macrophage colony-stimulating factor (GM-CSF) following CO exposure, which has previously been demonstrated in LPS-stimulated macrophages in vitro [32]. However we were unable to detect GM-CSF in either lung lavage fluid or plasma following LPS instillation (data not shown). Considering the absence of CO-induced changes in CXC chemokine production within this and other studies, these ‘non-specific’ effects of inhaled CO on neutrophil mobilisation and pulmonary neutrophil sequestration may be the primary mechanism by which CO exerts its effects in response to intratracheal LPS.

We also investigated whether the observed attenuation in neutrophil infiltration and mobilisation following LPS was associated with signs of attenuated pulmonary barrier dysfunction. Unexpectedly, even at 100 ppm we found that inhaled CO led to increased, rather than decreased, pulmonary barrier permeability. There was a small but statistically significant exacerbation of lavage fluid protein 24 hours after LPS in animals exposed to 100 ppm CO, consistent with a recent study showing that 50 ppm CO alone induced small increases in protein levels in lavage fluid of rats [33]. This accumulation of protein within lavage fluid over 24 hours represents a combination of increased barrier permeability with the process of alveolar fluid clearance. Currently there is no consensus on the impact of CO on lung fluid clearance. One study using mouse (renal) epithelial cells demonstrated an enhancement of sodium channel activity following addition of a CO donor [34], which theoretically may imply an enhancement of fluid clearance following CO. In contrast, inhaled CO has been shown to impair alveolar fluid clearance in isolated perfused rabbit lungs [11], which would tend to dilute and thus underestimate the differences in lavage fluid protein levels observed. In order to more directly evaluate whether CO induced barrier permeability, we assessed the movement of a fluorescence-labelled albumin across the endothelial and epithelial barriers. This was done in animals exposed to CO only (i.e. no LPS) for 6 hours. The data indicate that 100 ppm CO alone increased pulmonary barrier permeability. While the clinical significance of such relatively small changes is unclear, these findings provide a caution of potential undesirable effects of CO within the lungs even at low doses, which may have previously been overlooked.

The reasons for the discrepancies between those studies suggesting limited benefits of inhaled CO (such as the current study and those involving human subjects) compared to many pre-clinical studies showing very impressive results are not known. We speculate that these may be related to either i) the etiology of pulmonary inflammation/injury, or ii) the dose of CO used, and subsequent levels of COHb achieved. Although one previous study noted efficacy of inhaled CO as low as 10 ppm in a model of systemic inflammation [35], the vast majority of in vivo studies have utilised inhaled CO of 250–500 ppm or higher [4], [6]–[8], [21], [28], [36]. Such doses have been justified on the basis that these concentrations are below that used for lung diffusion capacity measurements in humans (3000 ppm) [7]. However, this justification may need re-evaluation considering the observed CO-Hb association/dissociation kinetics in mice. We found that the in vivo association kinetics for CO-Hb in mice are much more rapid than in humans; COHb% in blood reached ∼45% within 1 hour of exposure to 500 ppm CO, whereas the same level would not be reached until ∼3 hours in humans [37]. Thus, a short exposure to CO would produce much higher COHb levels in mice than humans. Consistent with the reported in vitro half-life of mouse COHb of 30 minutes [38], which is much shorter than that of human COHb (∼5 hours) [39], we found that COHb levels were almost halved 20 minutes after discontinuation of CO in vivo. This rapid dissociation may have inadvertently led to significant underestimation of the true blood COHb levels in mice in previous studies.

Such species differences in CO-Hb association/dissociation kinetics may at least partly explain why human trials of inhaled CO have been less successful than pre-clinical models. In one trial of healthy volunteers, 500 ppm CO for 1 hour failed to attenuate LPS-induced systemic inflammation [13], but this was associated with blood COHb levels of only 7%, well below that expected in rodents with similar exposure times. For comparison, in the current study 50 ppm CO for 24 hours, which showed variable efficacy, induced a similar COHb of 7.6±1.1% (n = 3). Consistent with such speculation, a study in endotoxin infused pigs demonstrated that when CO was administered to produce a blood COHb concentration of 5% (which the investigators used specifically as a non-toxic dose), there was no beneficial effect to attenuate inflammation [12]. The precise concentrations of CO within the tissue necessary to exert cytoprotective effects are unclear, and the relationship between blood COHb and tissue concentration of CO is highly complicated [39]. If blood COHb levels indeed reflect the amount of CO delivered to peripheral tissues, it is possible that inhaled CO would only be efficacious in humans once COHb reaches ‘close to toxic’ levels comparable to those achieved in rodent studies (e.g. 15–30%).

In summary, the current data support the concept that inhaled CO is capable of reducing inflammation to a moderate degree in a mouse model of relatively slowly progressing pulmonary inflammation/barrier dysfunction induced by a low dose of intratracheal LPS. In an attempt to explore the potential therapeutic applicability of inhaled CO, we determined 100 ppm CO to be efficacious and with few obvious side-effects. However, even this dose seemingly led to enhanced pulmonary epithelial/endothelial permeability. It remains possible that lower doses of CO, administered in a more chronic and/or intermittent manner, may have some therapeutic benefit for pulmonary disorders, although our data indicate that species differences in COHb dissociation/association kinetics make extrapolation from previous pre-clinical studies to human therapies difficult, and must be carefully considered when designing clinical trials. Overall, our data suggest a word of caution against the use of inhaled CO for the clinical treatment of pulmonary inflammatory diseases, until such complexities are better understood.

## References

[1] Ryter SW, Alam J, Choi AM (2006). Heme oxygenase-1/carbon monoxide: from basic science to therapeutic applications.. Physiol Rev.

[2] Zhou Z, Song R, Fattman CL, Greenhill S, Alber S (2005). Carbon monoxide suppresses bleomycin-induced lung fibrosis.. Am J Pathol.

[3] Chapman JT, Otterbein LE, Elias JA, Choi AM (2001). Carbon monoxide attenuates aeroallergen-induced inflammation in mice.. Am J Physiol Lung Cell Mol Physiol.

[4] Dolinay T, Szilasi M, Liu M, Choi AM (2004). Inhaled carbon monoxide confers antiinflammatory effects against ventilator-induced lung injury.. Am J Respir Crit Care Med.

[5] Kohmoto J, Nakao A, Stolz DB, Kaizu T, Tsung A (2007). Carbon monoxide protects rat lung transplants from ischemia-reperfusion injury via a mechanism involving p38 MAPK pathway.. Am J Transplant.

[6] Fujita T, Toda K, Karimova A, Yan SF, Naka Y (2001). Paradoxical rescue from ischemic lung injury by inhaled carbon monoxide driven by derepression of fibrinolysis.. Nat Med.

[7] Otterbein LE, Mantell LL, Choi AM (1999). Carbon monoxide provides protection against hyperoxic lung injury.. Am J Physiol.

[8] Nemzek JA, Fry C, Abatan O (2008). Low-dose carbon monoxide treatment attenuates early pulmonary neutrophil recruitment after acid aspiration.. Am J Physiol Lung Cell Mol Physiol.

[9] Clayton CE, Carraway MS, Suliman HB, Thalmann ED, Thalmann KN (2001). Inhaled carbon monoxide and hyperoxic lung injury in rats.. Am J Physiol Lung Cell Mol Physiol.

[10] Ghosh S, Wilson MR, Choudhury S, Yamamoto H, Goddard ME (2005). Effects of inhaled carbon monoxide on acute lung injury in mice.. Am J Physiol Lung Cell Mol Physiol.

[11] Althaus M, Fronius M, Buchackert Y, Vadasz I, Clauss WG (2009). Carbon monoxide rapidly impairs alveolar fluid clearance by inhibiting epithelial sodium channels.. Am J Respir Cell Mol Biol.

[12] Aberg AM, Abrahamsson P, Johansson G, Haney M, Winso O (2008). Does carbon monoxide treatment alter cytokine levels after endotoxin infusion in pigs?. A randomized controlled study J Inflamm (Lond).

[13] Mayr FB, Spiel A, Leitner J, Marsik C, Germann P (2005). Effects of carbon monoxide inhalation during experimental endotoxemia in humans.. Am J Respir Crit Care Med.

[14] Bathoorn E, Slebos DJ, Postma DS, Koeter GH, van Oosterhout AJ (2007). Anti-inflammatory effects of inhaled carbon monoxide in patients with COPD: a pilot study.. Eur Respir J.

[15] Spoelstra EN, Ince C, Koeman A, Emons VM, Brouwer LA (2007). A novel and simple method for endotracheal intubation of mice.. Lab Anim.

[16] Wilson MR, Choudhury S, Goddard ME, O'Dea KP, Nicholson AG (2003). High tidal volume upregulates intrapulmonary cytokines in an in vivo mouse model of ventilator-induced lung injury.. J Appl Physiol.

[17] Choudhury S, Wilson MR, Goddard ME, O'Dea KP, Takata M (2004). Mechanisms of early pulmonary neutrophil sequestration in ventilator-induced lung injury in mice.. Am J Physiol Lung Cell Mol Physiol.

[18] O'Dea KP, Wilson MR, Dokpesi JO, Wakabayashi K, Tatton L (2009). Mobilization and margination of bone marrow Gr-1high monocytes during sub-clinical endotoxemia predisposes the lungs towards acute injury.. J Immunol.

[19] Wilson MR, Goddard ME, O'Dea KP, Choudhury S, Takata M (2007). Differential roles of p55 and p75 tumor necrosis factor receptors on stretch-induced pulmonary edema in mice.. Am J Physiol Lung Cell Mol Physiol.

[20] Reutershan J, Basit A, Galkina EV, Ley K (2005). Sequential recruitment of neutrophils into lung and bronchoalveolar lavage fluid in LPS-induced acute lung injury.. Am J Physiol Lung Cell Mol Physiol.

[21] Ryter SW, Kim HP, Nakahira K, Zuckerbraun BS, Morse D (2007). Protective functions of heme oxygenase-1 and carbon monoxide in the respiratory system.. Antioxid Redox Signal.

[22] Perkins GD, Chatterjie S, McAuley DF, Gao F, Thickett DR (2006). Role of nonbronchoscopic lavage for investigating alveolar inflammation and permeability in acute respiratory distress syndrome.. Crit Care Med.

[23] Leite-Junior JH, Garcia CS, Souza-Fernandes AB, Silva PL, Ornellas DS (2008). Methylprednisolone improves lung mechanics and reduces the inflammatory response in pulmonary but not in extrapulmonary mild acute lung injury in mice.. Crit Care Med.

[24] Ritter C, da Cunha AA, Echer IC, Andrades M, Reinke A (2006). Effects of N-acetylcysteine plus deferoxamine in lipopolysaccharide-induced acute lung injury in the rat.. Crit Care Med.

[25] Frevert CW, Huang S, Danaee H, Paulauskis JD, Kobzik L (1995). Functional characterization of the rat chemokine KC and its importance in neutrophil recruitment in a rat model of pulmonary inflammation.. J Immunol.

[26] Thom SR, Weaver LK, Hampson NB (2005). “Therapeutic” carbon monoxide may be toxic.. Am J Respir Crit Care Med.

[27] Foresti R, Bani-Hani MG, Motterlini R (2008). Use of carbon monoxide as a therapeutic agent: promises and challenges.. Intensive Care Med.

[28] Otterbein LE, Bach FH, Alam J, Soares M, Tao Lu H (2000). Carbon monoxide has anti-inflammatory effects involving the mitogen-activated protein kinase pathway.. Nat Med.

[29] Otterbein LE, Otterbein SL, Ifedigbo E, Liu F, Morse DE (2003). MKK3 mitogen-activated protein kinase pathway mediates carbon monoxide-induced protection against oxidant-induced lung injury.. Am J Pathol.

[30] Nakahira K, Kim HP, Geng XH, Nakao A, Wang X (2006). Carbon monoxide differentially inhibits TLR signaling pathways by regulating ROS-induced trafficking of TLRs to lipid rafts.. J Exp Med.

[31] Wang XM, Kim HP, Nakahira K, Ryter SW, Choi AM (2009). The heme oxygenase-1/carbon monoxide pathway suppresses TLR4 signaling by regulating the interaction of TLR4 with caveolin-1.. J Immunol.

[32] Sarady JK, Otterbein SL, Liu F, Otterbein LE, Choi AM (2002). Carbon monoxide modulates endotoxin-induced production of granulocyte macrophage colony-stimulating factor in macrophages.. Am J Respir Cell Mol Biol.

[33] Ghio AJ, Stonehuerner JG, Dailey LA, Richards JH, Madden MD (2008). Carbon monoxide reversibly alters iron homeostasis and respiratory epithelial cell function.. Am J Respir Cell Mol Biol.

[34] Wang S, Publicover S, Gu Y (2009). An oxygen-sensitive mechanism in regulation of epithelial sodium channel.. Proc Natl Acad Sci U S A.

[35] Sarady JK, Zuckerbraun BS, Bilban M, Wagner O, Usheva A (2004). Carbon monoxide protection against endotoxic shock involves reciprocal effects on iNOS in the lung and liver.. Faseb J.

[36] Mazzola S, Forni M, Albertini M, Bacci ML, Zannoni A (2005). Carbon monoxide pretreatment prevents respiratory derangement and ameliorates hyperacute endotoxic shock in pigs.. Faseb J.

[37] Hoetzel A, Dolinay T, Schmidt R, Choi AM, Ryter SW (2007). Carbon monoxide in sepsis.. Antioxid Redox Signal.

[38] Watson ES, Jones AB, Ashfaq MK, Barrett JT (1987). Spectrophotometric evaluation of carboxyhemoglobin in blood of mice after exposure to marijuana or tobacco smoke in a modified Walton horizontal smoke exposure machine.. J Anal Toxicol.

[39] Piantadosi CA (2002). Biological chemistry of carbon monoxide.. Antioxid Redox Signal.

